# Effect of oral plaque control on postoperative pneumonia following lung cancer surgery

**DOI:** 10.1111/1759-7714.13448

**Published:** 2020-04-27

**Authors:** Chunling Jia, Mingxia Sun, Weizhi Wang, Cuirong Li, Xibo Li, Xiaoying Zhang

**Affiliations:** ^1^ Department of Oral Medicine Qilu Hospital of Shandong University Jinan China; ^2^ Institute of Stomatology Shandong University Jinan China; ^3^ Department of Thoracic Surgery Qilu Hospital of Shandong University Jinan China; ^4^ Department of Geriatrics Qilu Hospital of Shandong University Jinan China

**Keywords:** Dental plaque, lung cancer, plaque control, postoperative pneumonia, surgery

## Abstract

**Background:**

There have been few studies on the relationship between oral status and postoperative pneumonia (POP) in patients with lung cancer, and whether improving their oral condition assists with a lower incidence of POP before lung cancer surgery remains controversial. This retrospective study was conducted by a stomatologist to assess the effect of controlling oral pathogenic bacteria of patients with lung cancer to prevent POP.

**Methods:**

A total of 235 patients with lung cancer who underwent lobectomy by open thoracotomy between July 2015 and December 2018 were selected and given the choice of being in the experimental or control group. A total of 122 participants in the experimental group received professional oral plaque control, and 113 participants in the control group did not receive plaque control. All clinical data of participants in both groups were retrospectively studied to determine the incidence of POP at the thirtieth day of discharge from hospital.

**Results:**

Eight in the experimental group and six in the control group were excluded from the study. It was found that four of 114 patients suffered from POP in the experimental group (incidence = 3.51%). A total of 17 of 107 patients in the control group had pulmonary infection (incidence = 15.89%). Odds ratio was 0.19. The incidence of POP in the experimental group was significantly lower than that of the control group (*P* < 0.05).

**Conclusions:**

Professional oral plaque control is associated with a lower incidence of POP following lung cancer surgery and is therefore a favorable factor for preventing POP, and should be carried out before the surgical treatment of lung cancer.

**Key points:**

Professional oral plaque control was associated with a lower incidence of POP following lung cancer surgery, and it is recommended this should be carried out before the surgical treatment of lung cancer.

## Introduction

Lung cancer is the leading cause of cancer mortality worldwide in both men and women.^1^Pulmonary resection remains the mainstay of curative treatment option for patients with lung cancer. Pulmonary complications following lung cancer surgery are a major barrier to early recovery. Of these complications, postoperative pneumonia (POP) is the main cause of death in lung cancer patients.^2^ There have been various studies which have reported many factors related to POP, such as diabetes, advanced age, smoking, chronic lung diseases, congestive heart failure, mechanical ventilation or intubation, antibiotic therapy, immunosuppression, a long preoperative stay, and prolonged surgical procedures.^3^, ^4^, ^5^, ^6^ However, the association between the oral status of patients with lung cancer and POP has been rarely studied, and whether improving patients' oral condition before lung cancer surgery remains controversial.^7^

The oral cavity directly connects with the respiratory tract. Food residues, saliva, saliva proteins, food proteins, fats, and carbohydrates in the oral cavity jointly form a particular biological environment in the oral cavity, which provides suitable conditions for the colonization of a variety of microorganisms. Diverse microorganisms colonizing in the oral cavity exist in the form of plaque biofilm. The term plaque is rather nonspecific, which may be supragingival or subgingival and may be adherent or nonadherent to teeth or tissue. Dental plaque, a tooth‐borne biofilm that initiates periodontal disease and dental caries may also influence the initiation and progression of pneumonia because of relocalization of the bacteria from the biofilm into the respiratory tract.^8^, ^9^, ^10^ One cubic millimeter of dental plaque contains about 100 million bacteria and may serve as a persistent reservoir for potential pathogens, both oral and respiratory bacteria. Authors have recently explored whether oral health management can reduce the risk of pneumonia, and have found that patients with poor oral hygiene and poor periodontal conditions have a significantly higher rates of pneumonia.^11^, ^12^ Pulmonary infection rates in the elderly and patients in intensive care units have reduced significantly after treatment to improve adverse oral environments.^13^ However, some authors have reported that there is no difference in the effect of oral health management in POP for esophageal cancer patients, but carry out oral health management when stomatitis occurs during preoperative chemotherapy.^14^ Therefore, oral health management may prominently reduce incidences of pneumonia, while oral plaques in the oral cavity, especially subgingival plaques in the periodontal pocket of periodontitis patients, may be a key source of pathogenic bacteria causing pulmonary infection.

Plaque control by a dentist for the oral cavity is an effective, reliable measure for removing oral plaques, eliminating pathogenic bacteria, and treating periodontal diseases. Its goal is to decrease the quantity of organisms below a critical mass and alter the composition of the remaining bacterial flora to one associated with health by direct removal of pathogenic microorganisms and their byproducts or removal of contributing factors such as calculus and overhanging restorations. The aim of this study was to evaluate the efficiency of controlling oral pathogenic bacteria of patients with lung cancer by a dentist on preventing POP.

## Methods

The Ethics committee of Qilu Hospital of Shandong University approved this study, and written informed consent was obtained from all patients.

All patients with lung cancer who underwent thoracotomy at Thoracic Surgery Department of Qilu Hospital of Shandong University between July 2015 and December 2018 were selected.

Inclusion criteria were as follows: (i) all participants were confirmed on imaging and pathological biopsy to have primary lung cancer without distant metastasis, and ready for lobectomy by open thoracotomy. Patients who had other conditions such as diabetes mellitus, hypertension, COPD and others that may have influenced operation effect were excluded from the study. Preoperative pulmonary function, each index of laboratory testing and EKG were normal. All participants had not received neoadjuvant chemotherapy or previous radiotherapy, had >10 natural teeth, were 18 years < ages <70 years, with no smoking during the study period.

Criteria for POP: patients with three or more of the following indicators were considered as having POP.^15^, ^16^ (i) Patients had a fever (temperature > 38°C) 72 hours after surgery or once more within 72 hours; (ii) increased white blood cell count (>12 × 10^9^/L–15 × 10^9^/L), or second increase (>10 × 10^9^/L) after it returned to normal; (iii) chest imaging showed consolidation or increasing patchy shadows of lung tissues; and (iv) patients coughed up purulent sputum, or were confirmed sputum culture‐positive. Patients who met four and one other criterion were considered to have POP.

Oral examinations were conducted by an experienced dentist. The assessments consisted of the presence and degree of gingival inflammation (gingivitis) and periodontitis and oral hygiene status. Gingival status was assessed by the gingival index (GI) of Löe and Silness. Periodontal clinical measurements were performed at six sites per tooth and included probing depth (PD), clinical attachment level (CAL), bleeding on probing (BOP), and suppuration (SUP). The oral hygiene status was assessed by the percentage of total dental surfaces with dental plaque (≤ 20% = good; > 20% = poor). Neural red solution was used to disclose the dental plaque.

Characteristics of patients are shown in Table 1.

**Table 1:** Characteristics of all participants in the study

Parameter	Experimental group (*n* = 114)	Control group (*n* = 107)
Age (years; mean ± SD)	55.3 ± 13.5	56.2 ± 12.7
Males (n) Females (n)	75 39	72 35
Oral health status Missing teeth (n; mean ± SD) PD (mm; mean ± SD) CAL (mm; mean ± SD) BOP (% site; mean ± SD SUP (% site; mean ± SD) GI = 0 (n) GI = 1 (n) GI = 2 (n) GI = 3 (n)	5.7 ± 1.5 5.4 ± 0.5 4.6 ± 0.6 2.4 ± 4.4 2.0 ± 3.4 15 13 20 17	5.6 ± 1.6 5.5 ± 0.6 4.5 ± 0.7 2.2 ± 4.5 2.1 ± 3.4 13 10 19 15
Good hygiene status, n (%) Poor hygiene status, n (%)	33 (28.9%) 81 (71.1%)	29 (27.1%) 78 (72.9)
Initial PFT, mean ± SD FVC (% pred) FEV1 (% pred) FEV1/FVC >80%, n (%) DLCO (% pred) DLCO >80% pred, n (%)	101.7 ± 16.4 103.6 ± 20.3 114 (100%) 97.2.0 ± 17.1 114 (100%)	102.1 ± 15.9 102.9 ± 19.7 107 (100%) 96.7 ± 16.4 107 (100%)
Smoking Ever‐smoker, n (%) Pack‐year, mean ± SD	71 (62.3%) 42.1 ± 27.2	66 (61.7%) 41.9 ± 26.7
Charlson comorbidity score, n (%) Score 0–1 Score 2 Score > 2	64 (56.1%) 50 (43.9%) 0	60 (56.1%) 47 (43.9%) 0
See dentist (hardly), n (%)	108 (94.7%)	100 (93.5%)
Days of drain tube, mean ± SD	4.9 ± 2.1	5.0 ± 2. 0
Days in hospital, mean ± SD	21 ± 6.9	20.9 ± 7.1

PD, probing depth; CAL, clinical attachment level; BOP, bleeding on probing; SUP, suppuration; GI, gingival index; FVC, forced vital capacity; FEV1, forced expiratory volume; DLCO, diffusing capacity for carbon monoxide. Every parameter with mean ± SD has no significant difference between the groups by *t*‐test (*P >* 0.05).

A total of 235 patients were selected and given the choice of being included in the experimental or control group. A total of 122 participants in the experimental group received oral plaque control, and 113 participants in the control group did not receive plaque control.

Participants in the experimental group received professional oral plaque control by a dentist five days before lung cancer surgery. The same dentist was responsible for giving participants an oral examination, oral health education tailored to the specific situation, removal of some teeth with no reserve value, filling of cavities, and temporary fixation of loose teeth. Full‐mouth scaling, root planning, and polishing were performed so that they were brought to a specific state of health with no gingival bleeding and no dental plaque. During the perioperative period, following treatment at the Stomatology department, patients were asked to brush their teeth using the Bass method three times a day. Teeth were brushed for half an hour after each meal which lasted more than three minutes so that every tooth could be brushed effectively. Dental floss and toothpicks were used for interdental surface cleaning. Mouthrinse (10 mL 0.12% chlorhexidine was recommended) was used twice a day (once in the morning and evening). A dental examination was performed on the day before the patients were discharged from hospital, any factors which may affect oral plaque control was eradicated at the visit.

Participants in the control group did not receive any special oral plaque control and followed their routine daily oral procedures.

Clinical data of all participants in both groups were collected retrospectively at the thirtieth day of discharge from hospital (Tables 1 and 2).

**Table 2:** The incidence of postoperative pneumonia (POP) of all participants in the study

Groups	POP n (%)	*X* ^*2*^	OR (Odds ratio)	*P*‐value
Experimental group (*n* = 114) Control group (*n* = 107)	4 (3.51%) 17 (15.89%)	9.84	0.19	*0.0017

*Significant difference between groups (*P* < 0.05).

## Statistical analysis

All data were analyzed using Stata15.1 software. Continuous variables were expressed as mean ± standard deviation. The *t*‐test was used to analyze continuous variables. The *x* ^*2*^ test was used to compare categorical variables between groups. A *P*‐value < 0.05 was considered statistically significant.

## Results

Among the 122 participants in the experimental group, three were excluded as they failed to perform oral plaque control. Five participants in the experimental group and six in the control group were excluded for one of the following reasons: surgery time > four hours,^17^ the drainage tube could not be removed at the seventh day after surgery, lobectomy was changed to sublobar resection or sleeve resection, and complications such as bronchial stump fistula and anastomotic fistula. All participants were evaluated 30 days postoperatively. It was found that four of 114 patients suffered from POP in the experimental group (incidence = 3.51%). A total of 17 of 107 patients in the control group had pulmonary infection (incidence = 15.89%). The odds ratio was 0.19. The incidence of POP in the experimental group was significantly lower than that of the control group (*P* < 0.05) (Table 2).

## Discussion

There have been many studies published on the mechanism and prevention measures of POP.^18^, ^19^, ^20^, ^21^, ^22^ However, the incidence of POP of lung cancer patients with open thoracotomy has not decreased significantly,^17^ and it remains difficult to predict who will develop POP after lung cancer surgery.^6^ One of the important reasons is that there was insufficient data collected in the study because most of the researchers identified the factors of POP through detailed review of clinical information which excluded the oral condition of patients. Guided by the medical models of MDT (multiple disciplinary team), this study discusses the incidence of POP in the context of effect of oral condition on systemic diseases.

Smoking is commonly recognized as an important risk factor for lung cancer. Initiation of smoking at early ages leads to higher exposure and accumulation of smoking‐related toxins and, consequently, higher incidence of lung cancer.^4^ Smoking is also a high risk factor for periodontal diseases, especially severe periodontitis. Smokers have a higher prevalence of periodontitis than non‐smokers and, in this case, disease progression is more significant.^23^ To the best of our knowledge, the oral hygiene and health of lung cancer patients has not previously been reported in the literature. There were approximately 62% of patients in our study with a history of smoking, and these patients rarely visit dentists (94%±), meaning it is logical to infer that most lung cancer patients have poor oral health and a high incidence of periodontitis. This inference was confirmed during oral examination of all participants. Therefore, pneumonia is more likely to be induced in lung cancer patients following surgery.^24^ However, little research correlating the effect of oral pathogenic bacteria removal by professional plaque control on POP in patients with lung cancer has been reported.^20^, ^25^

The oral plaque biofilms are colonized with various flora related to pneumonia, such as *A. actinomycetemcomitans*, *P. gingivalis*, and *Fusobacterium* species among typical oral flora, and anaerobic bacteria in the periodontal pocket of periodontitis patients, including *Actinomyces israelii*, *Capnocytophaga* spp., *Eikenella corrodens*, *Prevotella intermedia*, and *Streptococcus constellatus*.^26^ These respiratory pathogenic bacteria, particularly lichen in the subgingival dental plaque of periodontal pocket of periodontitis patients, are seldom eliminated through the application of prophylactic antibiotics following thoracotomy.The failure is due to the fact that it is not possible to achieve effective antirespiratory pathogenic bacteria concentrations of antibiotics in saliva and dental plaque.^27^ Dental plaque, which adheres tightly to and between teeth, cannot be easily washed away by vigorous rinsing or water sprays. It also resists disruption by antimicrobial agents that cannot easily penetrate the protective polysaccharide matrix barrier characteristic of biofilms. However, dental plaque can be removed efficiently and controlled by professional and daily oral hygiene procedures. Professional interventions such as scaling, root planning, and polishing can remove mineralized plaque which has become dental calculus. Thus, if dental plaque is removed, the plaque‐harboring respiratory pathogenic microbes and cytokines and enzymes induced would be eradicated, and the microniches they regather would be destroyed. With the help of daily home mechanical intervention such as brushing of teeth, flossing and mouthrinse containing antimicrobial agents, plaque‐harboring pathogenic microbes would be under tighter control and prevented from reforming. Chlorhexidine,^23^ used in the present study, is one of the most effective antiplaque antimicrobial agents. It can reach a maximum depth of 1 to 2 mm in the subgingival area and destroy bacterial niches in the oral cavity. Microorganisms on the dorsal tongue, tonsil, and buccal mucosa can be rinsed away. It can also be a short‐term measure for use in patients who are temporarily unable to brush their teeth for any reason.

This study showed that the patients in the experimental group underwent oral plaque control, and that the incidence of POP was 3.51%. This data is similar to other research reported in the literature,^20^, ^24^ which concluded that POP was significantly lower than that in the control group without plaque control (15.89%) with a range of 12% to 17.5% as previously reported.^29^ The study shows that professional oral plaque control was associated with lower incidence of POP following lung cancer surgery, which is a favorable factor for preventing POP (OR = 0.19).

Two participants in the experimental group stopped rinsing their mouths with mouthwash more than two days and one did not brush their teeth using the method of Bass, and were subsequently considered as having failed to control oral plaque. They all did not suffer from POP. It is difficult to evaluate the influence of their failed plaque control. Therefore, they were excluded from the study.

There are some limitations to our study. First, our sample was smaller; thus a greater sample is required to evaluate the effect of oral health status on POP. Second, pathogenic bacteria in the airway cannot be controlled which could have influenced the percentage of POP in our study. Third, because groups were assigned according to patients' subjective wishes in this study, the results may not have accurately reflected the actual situation of all patients with lung cancer. These are insufficiencies in our research which should be further explored in the future.

In conclusion, professional oral plaque control was associated with a lower incidence of POP following lung cancer surgery. It is a favorable factor for preventing POP, and it is recommended that this procedure should be carried out before commencing the surgical treatment of lung cancer.

## Disclosure

No authors report any conflict of interest.
